# Association of anticardiolipin antibody and C677T in methylenetetrahydrofolate reductase mutation in women with recurrent spontaneous abortions: a new path to thrombophilia?

**DOI:** 10.1590/S1516-31802005000100004

**Published:** 2005-01-02

**Authors:** Egle Couto, Ricardo Barini, Renata Zaccaria, Joyce Maria Annicchino-Bizzacchi, Renato Passini, Belmiro Gonçalves Pereira, José Carlos Gama da Silva, João Luiz Pinto e Silva

**Keywords:** Methylenetetrahydrofolate reductase (NADPH_2_) Thrombophilia, Thrombosis, Antiphospholipid syndrome, Anticardiolipin antibodies, Metilenotetrahidrofolato redutase (NADPH2). Trombofilia, Trombose, Síndrome antifosfolipídica, Anticorpos anticardiolipina

## Abstract

**CONTEXT:**

Recurrent spontaneous abortion (RSA) has been associated with venous thrombosis in the mother. Acquired and inherited thrombophilia factors are possible causes.

**OBJECTIVE:**

To evaluate the association between thrombogenic factors and recurrent spontaneous abortion.

**TYPE OF STUDY:**

Case-control study.

**SETTING:**

Centro de Atenção Integral à Saúde da Mulher, Universidade Estadual de Campinas.

**METHODS:**

40 ml of blood was collected from 88 women attending an RSA clinic and 88 fertile women attending a family planning clinic, to evaluate the presence of acquired and inherited thrombophilia factors. Anticardiolipin antibodies (ACA), lupus anticoagulant and deficiencies of proteins C and S and antithrombin III were evaluated by enzyme-linked immunosorbent assay (ELISA), dilute Russell Viper Venom time (dRVVT), coagulometric and chromogenic methods. DNA was amplified by the polymerase chain reaction (PCR) to study factor V Leiden and G20210A mutations in the prothrombin gene and C677T mutation in the methylenetetrahydrofolate reductase (MTHFR) gene. Data were analyzed using odds ratios and a regression model for age adjustment. Fisher’s exact test was used to evaluate statistical relationships between associated factors and RSA.

**RESULTS:**

ACA was detected in 11 women with RSA and one fertile woman. Heterozygous C677T was detected in 59 women with RSA and 35 fertile women. Concomitant presence of ACA and C677T was found in eight women with RSA and no fertile women (p < 0.01).

**DISCUSSION:**

The meaning of the association between C677T mutation in the MTHFR gene and ACA is still not clear. It is possible that an inherited factor that alone would not strongly predispose a woman to thrombosis could, when associated with an acquired factor, start the process and increase the likelihood of thrombosis expression.

**CONCLUSIONS:**

ACA and C677T in the MTHFR gene are statistically associated with RSA. The association of these two conditions is a new finding in thrombogenic factors and RSA.

## RESUMO

**CONTEXTO:**

O aborto espontâneo recorrente já foi associado a vários fatores etiológicos e à trombose materna. Fatores de trombofilia adquiridos e hereditários podem ser causas destes eventos.

**OBJETIVO:**

Avaliar a associação entre fatores de trombofilia e aborto espontâneo recorrente.

**TIPO DE ESTUDO:**

Caso-controle.

**LOCAL:**

Centro de Atenção Integral à Saúde da Mulher, Universidade Estadual de Campinas, São Paulo, Brasil.

**MÉTODOS:**

Foram retirados 40 ml de sangue de 88 mulheres com aborto espontâneo recorrente do Ambulatório de Aborto Recorrente e 88 mulheres férteis do Ambulatório de Planejamento Familiar, pareadas por idade e raça, para pesquisar fatores de trombofilia adquiridos e hereditários. O anticorpo anticardiolipina (ACA), anticoagulante lúpico (LA) e a deficiência das proteínas C, S e antitrombina III foram pesquisados por ELISA (enzyme-linkek immunosorbent assay), dRVVT (dilute Russel Viper Venom Time, dRVVT) e métodos coagulométrico e cromogênico. O ácido desoxirribonucléico (DNA) foi amplificado pela técnica de reação em cadeia da polimerase (PCR) para o estudo das mutações fator V de Leiden, G20210A no gene da protrombina e C677T no gene da enzima metilenotetrahidrofolato redutase (MTHFR).

**RESULTADOS:**

O ACA foi encontrado em 11 mulheres com RSA e em uma mulher fértil [OR 12.4 (IC 95% 1.5 a 98.5)]. A mutação C677T heterozigota foi encontrada em 59 mulheres com RSA e em 35 mulheres férteis [OR 3.1 (IC 95% 1.7 a 5.7)]. A presença concomitante do ACA e da mutação C677T heterozigota foi encontrada em oito mulheres com aborto espontâneo recorrente e em nenhuma mulher fértil (p < 0,01).

**DISCUSSÃO:**

O significado da associação entre a mutação heterozigota C677T no gene da MTHFR e o anticorpo anticardiolipina não está claro. Pode-se supor que um fator hereditário que, isolado, não predisporia fortemente um indivíduo à trombose poderia, em associação com um fator adquirido, deflagrar o processo e intensificar a expressão da trombose.

**CONCLUSÕES:**

O ACA e a mutação C677T heterozigota no gene da MTHFR apresentaram associação estatística com RSA. A concomitância destas duas alterações é um novo achado no estudo de fatores trombogênicos em aborto espontâneo recorrente.

## INTRODUCTION

Recurrent spontaneous abortion (RSA) has been associated with many etiologies. It occurs in 1 to 2% of all women of reproductive age.^1^ An association between autoantibodies and gestational loss has been identified, especially for antiphospholipid antibodies (APA). Other antibodies such as those against thyroid components, nuclear antigens and deoxyribonucleic acid (DNA) fractions^2^ have also been associated with gestational loss and preeclampsia.^3^

Venous thrombosis in the mother has been associated with RSA in several series.^4,5^ Acquired thrombophilia is associated with thrombophilic factors with no inherited characteristics, such as anticardiolipin antibody (ACA) and lupus anticoagulant (LA).

The detection of APA such as ACA and LA seems to be higher in the first trimester in women who have antiphospholipid syndrome (APS), but positive transient results have been detected in women without APS. APS and implantation site thrombosis can justify 5 to 10% of RSA and the mechanism of action is not completely clear.^6^

LA is an immunoglobulin that interferes with one or more phospholipiddependent coagulation tests and leads to thromboembolic predisposition.^7^ Patients with persistent elevated results are at risk of arterial and venous thrombosis, gestational loss and other complications, such as intrauterine growth restriction.^5^ Simultaneous presence of LA and other APA seems to have an important association with early abortion.^8^ Inherited factors for thrombophilia include protein C, protein S and antithrombin III (ATIII) deficiencies, factor V Leiden and G20210A mutations in the prothrombin gene and C677T mutation in the methylenetetrahydrofolate reductase (MTHFR) gene.

Other APA have also been associated with RSA, such as antiphosphatidylserine and antiphosphatidylethanolamine. Some of these antibodies are directed to phosphatidyl ethanolamine-binding plasma proteins, such as high molecular weight kininogen.^9^ The kininogen concentration in reproductive tissues and plasma has been reported to fluctuate during ovulation, pregnancy and parturition.^10^ What governs the fluctuation of kininogen concentrations remains to be elucidated. Sugi et al.^9^ demonstrated a strong association between RSA and antiphosphatidylethanolamine antibodies. Studying women with recurrent in vitro fertilization failure, Matsubayashi et al.^11^ found an increased incidence of IgG-antiphosphatidyl-serine, when compared with RSA patients.

Markers of deficiency in folate metabolic pathways have been associated with RSA, including elevated levels of plasmatic homocysteine.^12,13^ Several inherited and acquired conditions can cause hyperhomocysteinemia,^14^ leading to increased risk of stroke, myocardial infarction and peripheral arterial disease.^15^ The C677T mutation in the MTHFR gene leads to a reduction in the activity of this enzyme, resulting in increased levels of plasmatic homocysteine.^16^ Several series have suggested an association between hyperhomocysteinemia and RSA.^12,13^ Other gestational complications, such as preeclampsia, abruptio placentae, intrauterine growth restriction and stillbirth have been associated with homozygous C677T in the MTHFR gene.^17^

Proteins C and S are natural plasmatic anticoagulants.^15^ The prevalence of protein C deficiency in women with gestational loss ranges from 4.7%^13^ to 25.5%.^18^ A high prevalence of protein S deficiency has been found in women with two or more spontaneous abortions: 44.8%. ATIII deficiency has also been associated with first trimester abortions^18^ and preeclampsia.^19^

A mutation in the factor V gene has been identified as the molecular basis for activated protein C (aPC) resistance and has been named factor V Leiden. This leads to a hypercoagulable state.^20^ Dizon-Townson et al.^21^ did not detect any no association between RSA and factor V Leiden when studying women with RSA and fertile women. Despite this, a clear association between RSA and factor V Leiden was detected by Foka et al.^22^

The G to A transition at the 20210 position in the prothrombin gene is associated with elevated prothrombin levels and is a risk factor for thrombosis.^23^ Studying 80 women with RSA and 100 fertile women, Foka et al.^22^ detected a higher frequency of preeclampsia, abruptio placentae, intrauterine growth restriction and stillbirth among those who were carriers of this mutation.

It is well established that inherited or acquired thrombophilic factors and their combinations can lead to increased risk of thrombosis.^6^ These combinations have also been associated with 2.2 to 14.3 times greater risk of RSA.^24^ The objective of the present study was to evaluate the association between the presence of thrombophilic factors and RSA.

## METHODS

This was a case-control study that included 88 women with RSA who were sequentially attending an RSA clinic at Universidade Estadual de Campinas (Centro de Atenção Integral à Saúde da Mulher) as outpatients (Group 1) and 88 fertile women with at least one successful pregnancy who were attending a family planning clinic at the same institution (Group 2), from January 1999 to August 2000. The women in Group 2 had no history of RSA, stillbirth, thromboembolic episodes, arterial hypertension, bad obstetrics or low birth weight. The patients in Group 1 were matched with the controls in Group 2 by age (five-year groups) and race (white or non-white). 40 ml of blood was collected to evaluate the presence of acquired (ACA and LA) and inherited factors (protein C, protein S and ATIII deficiencies, factor V Leiden and G20210A mutations in the prothrombin gene and C677T mutations in the MTHFR gene).

Enzyme-linked immunosorbent assay (ELISA) was performed as previously described,^25^ using high positive controls with known immunoglobulin G (IgG) and immunoglobulin M (IgM) concentrations (calibration sera LAPL-MP-005 and LAPL-GP-005 from Louisville, Kentucky) and a normal donor as the negative control, in order to detect ACA. For quantitative determination of ACA, ELISA was used according to the manufacturer’s instructions and the standard ELISA procedures. In-house calibrators were used for standardization. Negative controls were included in the assays to confirm negative test results. These negative controls consisted of negative serum tested in our laboratory according to standard procedures. Positive test results for patients were selected by means of positive controls from Louisville. The absorbance was determined using a photometer set at 450 nm. The cutoff values predefined by the manufacturers were: IgG > 20 units and IgM > 20 units. The interassay variation was 2.9-7.4% for IgG and 2.3-5.1% for IgM. Mean values were also determined, and the results were compared using multiples of the mean.

LA was detected by the dilute Russell Viper Venom Time (dRVVT), using the Organon Teknika kit: Viperquik^TM^ LA-test and Viperquik^TM^ LA-check. The final result was expressed as a relationship between "test" and "check" results. When this ratio was greater than 1.20, a confirmatory test was performed using a 50% mixture. Results greater than 2.0 were considered positive.

Protein S (PS) activity was evaluated via the coagulometric method using the Bioclot PS kit. Normal diluted plasma was mixed with PS-deficient plasma. This mixture was activated by activated factor X + activated protein C + phospholipids. After the addition of calcium chloride, the prolongation of clotting time was considered to be proportional to the protein S concentration in the patient’s plasma. Normal activity ranged from 55% to 160%.

Protein C activity was evaluated by the prolongation of the activated partial thromboplastin time (APTT), using southern copperhead snake poison to activate it. The protein C assay was performed using the Dade kit from Baxter Diagnostics, Inc. Normal activity ranged from 72% to 142%.

ATIII functional levels were determined using plasma diluted with ATIII, heparin and excess thrombin. An ATIII-thrombin-heparin complex was formed, and the remaining thrombin catalyzed p-nitroaniline releases on a chromogenic substrate. These were measured at 405 nm and viewed on a calibration curve. The Chromostrate^®^ kit from Organon Teknika was used, and normal values ranged from 85% to 125%.

Genomic DNA was extracted from peripheral blood by a standard method.^26^ The polymerase chain reaction (PCR) was used to assay the factor V Leiden mutation, the thermolabile MTHFR C677T mutation and the prothrombin G20210A mutation. Amplification of factor V Leiden was performed using a mixture of 54 mM Tris HCl (pH 8.8), 5.4 mM MgCl_2_, 5.4 µM EDTA, 13.3 mM (NH_4_)_2_ SO_4_, 8% DMSO, 8 mM β-mercaptoethanol, 0.4 mg/ml BSA, 0.8 mM of each nucleoside triphosphate, 200 ng of each forward and reverse primer, 250 to 500 ng of genome DNA and 2U Taq polymerase. The amplification was performed for 36 cycles of 91° C (40 seconds), 55° C (40 seconds) and 71° C (two minutes).

For MTHFR and prothrombin, amplifications were performed in separate 50 µl reactions containing 10 mM Tris HCl (pH 8.3), 50 mM KCl, 1.5 mM MgCl, 0.2 mM of each nucleoside triphosphate, 0.4 µM of each forward and reverse primer and 2.5U of Taq polymerase. The PCR parameters were 38 cycles of 94° C (30 seconds), 54° C (30 seconds) and 72° C (30 seconds). The initial cycle was preceded by 9 minutes at 94° C, so as to activate AmpliTaq Gold polymerase in addition to denaturing the template, and the last cycle was followed by 5 minutes at 72° C. The PCR products were digested with the appropriate restriction enzyme. After digestion, PCR products were submitted to electrophoresis on 2% agar minigels containing ethidium bromide at 120 V for 1 hour.

For factor V Leiden, MnII digestion of the 267-bp PCR product yielded fragments of 163, 67 and 37 bp for the normal allele. The digestion products of the mutant allele were 200 and 67 bp. For MTHFR, HynfI did not cleave the 198-bp PCR product of the normal allele, whereas the mutant allele yielded fragments of 175 bp and 23 bp after HinfI digestion.

For prothrombin, HindIII digestion yielded intact 345-bp product for the normal allele and two fragments of 322 and 23 bp for the mutant allele. For each locus, heterozygous individuals exhibited both normal and mutant digestion products. The PCR assay controls included DNA from a subject with subject, a normal subject and a blank water run for each analysis.

The data were analyzed using the odds ratio (OR) and a regression model for ageadjustment. Fisher’s exact test was used to evaluate statistical relationships between RSA and the associated factors. The results were considered statistically significant when *p* values were less than 5%, for alpha = 0.05 and beta = 0.20.

The ethical principles of the Helsinki Declaration of the World Medical Association were followed in this study. The Ethics Committee of Universidade Estadual de Campinas, Brazil, approved this study.

## RESULTS

The mean age was 30.4 years for Group 1 and 29.7 for Group 2. The total number of pregnancies for patients from Group 1 was 346, which ended as 26 deliveries and 320 spontaneous abortions. In Group 2, the total number of pregnancies was 181, and all of them ended as deliveries. In each group studied, 61.4% of the women were white. In Group 1, 55.7% had had three pregnancies and 44.3% had had four or more. In Group 2, 36.4% had had just one pregnancy. In Group 1, 64.8% had had three gestational losses and 35.2% had had four or more losses. ACA was detected in eleven women from Group 1 and in one woman from Group 2 (OR 12.4; 1.5 to 98.5). The C677T mutation in the MTHFR gene was detected in 59 women from Group 1 and 35 from Group 2 (OR 3.1; 1.7 to 5.7). These results can be observed in Table 1.

**Table 1:** Thrombophilic factor distribution in 88 women with recurrent abortion (Group 1) and 88 fertile women (Group 2) attended at an outpatient clinic in Brazil

	Group 1		Group 2		OR (95% CI)	Age-adjusted OR (1) (95% CI)
	n	(%)	n	(%)		
Anticardiolipin antibodies	11	(12.5)	1	(1.1)	12.4 (1.5 to 98.5)	12.9 (1.5 to 109.5)
Lupus anticoagulant	1	(1.1)	0	(0)	–	–
Protein S deficiency	1	(1.1)	0	(0)	–	–
Protein C deficiency	2	(2.3)	0	(0)	–	–
AT III deficiency	1	(1.1)	0	(0)	–	–
Factor V Leiden	3	(3.4)	0	(0)	7.2 (0.3 to 142.4)	–
G20210A prothrombin	1	(1.1)	1	(1.1)	1.0 (0.06 to 16.2)	1.0 (0.04 to 22.8)
C677T mutation in MTHFR gene	59	(67.0)	35	(39.8)	3.1 (1.7 to 5.7)	3.3 (1.6 to 6.6)

*(1) Age adjustment was performed using a multiple regression model.*

*OR = odds ratio; CI = confidence interval; MTHFR = methylenetetrahydrofolate reductase.*

Table 2 shows the distribution of homozygous and heterozygous C677T mutation in the two groups. A statistically significant difference was found when women with heterozygous mutation were compared with normal women.

**Table 2:** Homozygous and heterozygous C677T mutation in the methylenetetrahydrofolate reductase gene in 88 women with recurrent abortion (Group 1) and 88 fertile women (Group 2) attended at an outpatient clinic in Brazil

C677T mutation in MTHFR gene	Group 1		Group 2		p
	(n)	%	(n)	%	
Homozygous, TT [a]	12	13.6	9	10.2	a versus c = 0.06
Heterozygous, CT [b]	47	53.4	26	29.5	b versus c = 0.0003
Normal, CC [c]	29	33.0	53	60.2	
Total	88	100.0	88	100.00	

3 *MTHFR = methylenetetrahydrofolate reductase.*

Table 3 shows the allele distribution of C677T mutation between the groups studied. The mutant allele (T) was found in 40.3% of the women in Group 1 and in 25% of the women in Group 2.

**Table 3:** Methylenetetrahydrofolate reductase gene allele distribution in 88 women with recurrent abortion (Group 1) and 88 fertile women (Group 2) attended at an outpatient clinic in Brazil

Allele	Group 1		Group 2		p
	(n)	%	(n)	%	
Mutant (T)	71	40.3	44	25.0	0.002
Normal (CC)	105	59.7	132	75.0	
Total	176	100.0	176	100.00	

No statistically significant differences were detected in the distribution of thrombophilic factors when patients were divided into white and non-white skin color.

The analysis of associated factors and their comparison between the groups studied showed that ACA and the heterozygous C677T mutation in the MTHFR gene were simultaneously present in eight women from Group 1 and no one from Group 2, and this difference was statistically significant. The association between ACA and the homozygous C677T mutation in the MTHFR gene showed no statistical difference between the two groups; nor did the associations between ACA and the other thrombophilic factors studied. These relationships can be seen in Table 4.

**Table 4:** Associations between anticardiolipin antibodies and other thrombophilic factors: distribution in 88 women with recurrent abortion (Group 1) and 88 fertile women (Group 2) attended at an outpatient clinic in Brazil

	Factor associations	Group 1		Group 2		p*
		(n)	%	(n)	%	
ACA with:	Lupus anticoagulant	0	0	0	0	1.00
	Protein C deficiency	1	1.1	0	0	1.00
	Protein S deficiency	0	0	0	0	1.00
	ATIII deficiency	1	1.1	0	0	1.00
	Leiden factor V	0	0	0	0	1.00
	G20210A prothrombin	0	0	0	0	1.00
	Heterozygous C677T	8	9.1	0	0	< 0.01
	Homozygous C677T	0	0	0	0	1.00
**Total**		**(10)**	**(11.3)**	**(0)**	**(0)**	

* *Fisher’s exact test. ACA = anticardiolipin antibody.*

Likewise, the associations between the heterozygous C677T mutation in the MTHFR gene and the other factors studied showed no statistically significant difference between the two groups. The homozygous C677T mutation in the MTHFR gene showed no association with any thrombophilic factor studied.

## DISCUSSION

In this study we looked for associations of inherited and acquired thrombophilic factors among patients with and without recurrent spontaneous abortions. Patients were matched by age, in order to try to minimize any possible unknown effect of age variation on RSA, or any possible effect of acquired factors (ACA and LA) on older women, regardless of the fact that no differences in the frequencies of these antibodies have been described for several age groups.^27^

It is known that the prevalence of genetic diseases varies in different races. As the Brazilian population is highly heterogeneous, patients were also matched by race. A higher prevalence of factor V Leiden among Caucasians has been described.^28^ It is possible that other mutations also present higher prevalence in this racial group. In the Brazilian population, a higher prevalence of factor V Leiden has been detected in Caucasians, and it has rarely been detected in black people and Indians.^29^

The mean number of pregnancies was higher in Group 1. This result was expected, since the definition of RSA includes women with at least three spontaneous abortions. 36.4% of the women in Group 2 were primiparous. Although we could not know how the next pregnancies would develop for this group, primiparous women were included as participants in the control group, in the same way as previously described in other series.^30,31^ After one successful pregnancy, approximately 3% of women will experience a spontaneous abortion.^1^ Considering this probability, only one woman from Group 2 would be expected to have this complication and this proportion would not be enough to change our results.

ACA positivity occurred in 12.5% of the women who had RSA, and this coincides with the results in the literature.^5^ Among fertile women, it was detected in 1.1%. The prevalence of ACA described in normal obstetric populations ranges from 2.9 to 3.6%.^32^

LA was detected in one patient from Group 1 and none from Group 2. Several series have been described that show an association between LA and RSA.^5,8^ However, LA levels can vary during pregnancy,^7^ and persistently elevated levels are strongly associated with gestational loss.^5^ We did not find a good correlation between this test and RSA in this group of patients. There is a chance that this factor is not an important one for our population.

The prevalence of deficiencies of protein C, protein S and ATIII did not differ between the groups studied. A higher risk of spontaneous abortion in women with these deficiencies has been reported.^33^ Since protein C deficiency can affect 10 to 15% of young individuals with recurrent venous thrombosis^20^ and protein S deficiency occurs in 2.2% of patients with venous thrombosis,^15^ we investigated whether thrombosis at an implantation site could lead to recurrent abortion in women with these deficiencies. This hypothesis was not confirmed by our results. Two women from Group 1 and none from Group 2 had protein C deficiency. As the incidence of this deficiency in the general population is 1/33,000 individuals,^15^ a statistically significant difference might perhaps be detected with a greater number of patients.

Women of reproductive age who are deficient in protein C, protein S or ATIII have a three times higher risk of thromboembolic disease than do men of the same age.^34^ Women with serious effects from a stroke or myocardial infarction may have more difficulty becoming mothers, as much due to their clinical condition as due to restrictions on social relations. Thus, the evaluation of women with RSA could exclude serious cases in an involuntary manner, thereby modifying the results.

There were no statistically significant differences in the frequencies of factor V Leiden and G20210A mutation in the prothrombin gene between the two groups. The literature shows large variation in the importance of these mutations in relation to RSA. Some series have demonstrated an association between activated protein C resistance and abortions,^30^ but the picture is not so clear when trying to implicate factor V Leiden as responsible for this resistance in the abortion series.^35^

Some authors have confirmed an association between factor V Leiden and RSA, but others have not, as can be seen in Table 5. Our results did not demonstrate such an association. Although three women from Group 1 were detected as factor V Leiden carriers, there were none in Group 2. Again, the use of a larger sample size would perhaps give rise to a significant difference between the two groups.

**Table 5:** List of some series with or without an association between factor V Leiden and recurrent spontaneous abortion

Author	Year	Cases	Controls	p
Rai et al.^30^	1996	70	70	0.02
Balash et al.^31^	1997	55	50	NS
Dizon-Townson et al.^21^	1997	40	25	NS
Grandone et al.^37^	1997	43	118	0.011
Pauer et al.^38^	1998	84	84	NS
Kutteh et al.^39^	1999	50	50	NS
Souza et al.^40^	1999	56	384	0.03
Foka et al.^22^	2000	80	100	0.003
Wramsby et al.^41^	2000	84	69	0.024

*NS = not significant.*

G20210A carriers have higher prothrombin levels and are at greater risk of thrombosis than are controls.^23^ A higher risk of primary abortion was detected in women with this mutation than in controls.^22^ Our results, with a similar number of patients, did not show a significant difference in G20210A prevalence between the groups studied. More studies are necessary for a definitive conclusion about the importance of this mutation for RSA to be reached.

The evaluation of the C677T mutation in the MTHFR gene resulted in curious data. We observed an association of this mutation with RSA, but such an association is not completely clear in the literature. Some authors agree that C677T, as a cause of hyperhomocysteinemia, could lead to RSA,^17,22^ but others do not.^36,37^

Homozygous C677T was detected in twelve women from Group 1 and in nine from Group 2. This difference was not significant. The high number of women with homozygous C677T MTHFR who had successful pregnancies (nine women from Group 2) suggests that this mutation is not an essential condition for a bad gestational result. None of these nine women had ACA in association with homozygous C677T MTHFR.

Heterozygous C677T was found in 47 women from Group 1 and in 26 from Group 2, and this difference was statistically significant. These data do not agree with the literature. Some series in which C677T is classified as homozygous or heterozygous have shown a clear predominance of statistically significant homozygosity in RSA.^16,21^ Lissak et al.^38^ described the importance of heterozygous C677T in RSA, but stated that more series would be necessary to confirm this. Homozygous individuals have high homocysteine levels, but increased levels can also be seen in heterozygous individuals.

Nonetheless, the allele frequency of the C677T mutation in the MTHFR gene was higher in Group 1, with a statistically significant difference between the two groups. This may direct new investigations towards the importance of heterozygous C677T mutation in women with RSA.

The concept that several factors interact to create thrombosis is well established.^39^ The action of thrombophilic factors during placentation may become amplified, thereby giving rise to modifications in placental circulation. These events could lead to spontaneous abortion or other gestational complications, such as intrauterine growth restriction and stillbirth.

Several congenital and acquired factors must act together to suppress the potent antithrombotic mechanism.^39^ The association of ACA and heterozygous C677T mutation was detected in eight women from Group 1 and none from Group 2, and this difference was statistically significant.

The meaning of this association is still not clear. It is possible that an inherited factor that alone would not strongly predispose a woman to thrombosis could, when associated with an acquired factor, start the thrombosis process.

The association between heterozygous C677T mutation in the MTHFR gene and ACA may increase the likelihood of thrombosis expression. It is known that persistently elevated serum levels of ACA are associated with RSA.^40^ Positive results for ACA may be the starting point for the process of thrombosis. We believe that this kind of association may be the key to understanding the mechanisms for several diseases, such as RSA, cancer or cardiac infarction, and should be further investigated.

There is no evidence in the literature that this kind of association is absolutely necessary for the full manifestation of the antiphospholipid syndrome. However, it is possible that an inherited factor, such as C677T, may act as a genetic predisposing factor that interacts with the antiphospholipid antibodies to fully manifest their clinical aspects. Moreover, we cannot state whether such associations are the cause of the antiphospholipid syndrome or whether C677T is the inducer of anticardiolipin antibodies. But we can wonder whether this association might be necessary for ACA manifestation.

## CONCLUSIONS

ACA and heterozygous C677T mutation in the MTHFR gene presented a statistical association with RSA. The association of these two conditions is a new finding in thrombogenic factors for RSA, and may contribute to a greater understanding of this event.

## References

[1] Regan L, Braude PR, Trembath PL (1989). Influence of past reproductive performance on risk of spontaneous abortion. BMJ.

[2] Qureshi F, Yang Y, Jaques SM (2000). Anti-DNA antibodies crossreacting with laminin inhibit trophoblast attachment and migration: implications for recurrent pregnancy loss in SLE patients. Am J Reprod Immunol.

[3] Mecacci F, Parretti E, Cioni R (2000). Thyroid autoimmunity and its association with non-organ-specific antibodies and subclinical alterations of thyroid function in women with a history of pregnancy loss or preeclampsia. J Reprod Immunol.

[4] Hughes GR (1983). Thrombosis, abortion, cerebral disease and the lupus anticoagulant. Br Med J (Clin Res Ed).

[5] Triplett DA, Coulam CB, Faulk WP, McIntyre JA (1992). Obstetrical complications associated with antiphospholipid antibodies. Immunological obstetrics.

[6] Younis JS, Ohel G, Brenner B, Haddad S, Lanir N, Ben-Ami M (2000). The effect of thrombophylaxis on pregnancy outcome in patients with recurrent pregnancy loss associated with factor V Leiden mutation. BJOG.

[7] Lockshin MD, Qamar T, Levy RA, Scott JS, Bird HA (1990). Anticardiolipin and related antibodies: thrombosis and fetal death. Pregnancy, autoimmunity and connective tissue disorders.

[8] MacLean MA, Cumming GP, McCall F, Walker ID, Walker JJ (1994). The prevalence of lupus anticoagulant and anticardiolipin antibodies in women with a history of first trimester miscarriages. Br J Obstet Gynaecol.

[9] Sugi T, Katsunuma J, Izumi S, McIntyre JA, Makino T (1999). Prevalence and heterogeneity of antiphosphatidylethanolamine antibodies in patients with recurrent early pregnancy losses. Fertil Steril.

[10] Hossain AM, Whitman GF, Khan I (1995). Kininogen present in rat reproductive tissues is apparently synthesized by the liver, not by the reproductive system. Am J Obstet Gynecol.

[11] Matsubayashi H, Sugi T, Arai T (2001). Different antiphospholipid antibody specificities are found in association with early repeated pregnancy loss versus recurrent IVF-failure patients. Am J Reprod Immunol.

[12] Wouters MG, Boers GH, Blom HJ (1993). Hyperhomocysteinemia: a risk factor in women with unexplained recurrent early pregnancy loss. Fertil Steril.

[13] Coumans AB, Huijgens PC, Jakobs C (1999). Haemostatic and metabolic abnormalities in women with unexplained recurrent abortion. Hum Reprod.

[14] Nelen WL, Blom HJ, Steegers EA, den Heijer M, Thomas CM, Eskes TK (2000). Homocysteine and folate levels as risk factors for recurrent early pregnancy loss. Obstet Gynecol.

[15] De Stefano V, Finazzi G, Mannucci PM (1996). Inherited thrombophilia: pathogenesis, clinical syndromes, and management. Blood.

[16] Lane DA, Mannucci PM, Bauer KA (1996). Inherited thrombophilia: Part 1. Thromb Haemost.

[17] Kupferminc MJ, Eldor A, Steinman N (1999). Increased frequency of genetic thrombophilia in women with complications of pregnancy. N Engl J Med.

[18] Ogasawara MS, Aoki K, Katano K, Ozaki Y, Suzumori K (2001). Factor XII but not protein C, protein S, antithrombin III, or factor XIII is a predictor of recurrent miscarriage. Fertil Steril.

[19] Galstian A, Beer AE, Roberts JM, Coulam CB, Faulk WP, Coulam CB, Faulk WP, McIntyre JA (1992). Immunology of preeclampsia. Immunological obstetrics.

[20] Dahlback B (1995). The protein C anticoagulant system: inherited defects as basis for venous thrombosis. Thromb Res.

[21] Dizon-Townson DS, Kinney S, Branch DW, Ward K (1997). The factor V Leiden mutation is not a common cause of recurrent miscarriage. J Reprod Immunol.

[22] Foka ZJ, Lambropoulos AF, Saravelos H (2000). Factor V leiden and prothrombin G20210A mutations, but not methylenetetrahydrofolate reductase C677T, are associated with recurrent miscarriages. Hum Reprod.

[23] Poort SR, Rosendaal FR, Reitsma PH, Bertina RM (1996). A common genetic variation in the 3’-untranslated region of the prothrombin gene is associated with elevated plasma prothrombin levels and an increase in venous thrombosis. Blood.

[24] Blumenfeld Z, Brenner B (1999). Thrombophilia-associated pregnancy wastage. Fertil Steril.

[25] Triplett DA, Barna L, Unger G (1993). Laboratory identifications of lupus anticoagulants. XIVth Congress of the International Society on Thrombosis and Haemostasis.

[26] Sambrook J, Fritsch EF, Maniatis T (1989). Molecular cloning: a laboratory manual.

[27] Ruiz AM, Kwak JY, Kwak FM, Beer AE (1996). Impact of age on reproductive outcome in women with recurrent spontaneous abortions and infertility of immune etiology. Am J Reprod Immunol.

[28] Rees DC, Cox M, Clegg JB (1995). World distribution of factor V Leiden. Lancet.

[29] Arruda VR, von Zuben PM, Soares MC, Menezes R, Annichino-Bizzacchi JM, Costa FF (1996). Very low incidence of Arg506->Gin mutation in the factor V gene among the Amazonian Indians and the Brazilian black population. Thromb Haemost.

[30] Rai R, Regan L, Hadley E, Dave M, Cohen H (1996). Second-trimester pregnancy loss is associated with activated C resistance. Br J Haematol.

[31] Balasch J, Reverter JC, Fábregues F (1997). First-trimester repeated abortion is not associated with activated protein C resistance. Hum Reprod.

[32] Branch DW, Silver R, Pierangeli S, van Leeuwen I, Harris EN (1997). Antiphospholipid antibodies other than lupus anticoagulant and anticardiolipin antibodies in women with recurrent pregnancy loss, fertile controls, and antiphospholipid syndrome. Obstet Gynecol.

[33] Preston FE, Rosendaal FR, Walker ID (1996). Increased fetal loss in women with heritable thrombophilia. Lancet.

[34] Bucciarelli P, Rosendaal FR, Tripodi A (1999). Risk of venous thromboembolism and clinical manifestations in carriers of antithrombin, protein C, protein S deficiency, or activated protein C resistance: a multicenter collaborative family study. Arterioscler Thromb Vasc Biol.

[35] Dulícek P, Chrobák L, Kalousek I, Pesavová L, Pecka M, Stránsky P (1999). Is factor V Leiden a risk factor for fetal loss?. Acta Medica (Hradec Kralove).

[36] Ray JG, Laskin CA (1999). Folic acid and homocyst(e)ine metabolic defects and the risk of placental abruption, pre-eclampsia and spontaneous pregnancy loss: A systematic review. Placenta.

[37] Murphy RP, Donoghue C, Nallen RJ (2000). Prospective evaluation of the risk conferred by factor V Leiden and thermolabile methylenetetrahydrofolate reductase polymorphism in pregnancy. Arterioscler Thromb Vasc Biol.

[38] Lissak A, Sharon A, Fruchter O, Kassel A, Sanderovitz J, Abramovici H (1999). Polymorphism for mutation of cytosine to thymine at location 677 in the methylenetetrahydrofolate reductase gene is associated with recurrent early fetal loss. Am J Obstet Gynecol.

[39] Phillips MD (1997). Interrelated risk factors for venous thromboembolism. Circulation.

[40] Topping J, Quenby S, Farquharson R, Malia R, Greaves M (1999). Marked variation in antiphospholipid antibodies during pregnancy: relationships to pregnancy outcome. Hum Reprod.

